# Deformierende endonasale Raumforderung während der Schwangerschaft

**DOI:** 10.1007/s00106-023-01290-1

**Published:** 2023-03-22

**Authors:** A. P. Maas, J. Eckrich, T. Send, M. Bernhardt, S. Strieth, B. P. Ernst

**Affiliations:** 1 Klinik und Poliklinik für Hals-Nasen-Ohren-Heilkunde, Universitätsklinikum Bonn, Venusberg-Campus 1, 53127 Bonn, Deutschland; 2 Institut für Pathologie, Universitätsklinikum Bonn, Venusberg-Campus 1, 53127 Bonn, Deutschland

**Keywords:** Epistaxis, Lobuläres kapilläres Hämangiom, Endonasaler Tumor, Endoskopische Nasenchirurgie, Granuloma gravidarum, Epistaxis, Lobular capillary hemangioma, Endonasal tumor, Endoscopic sinunasal surgery, Granuloma gravidarum

## Abstract

Beschrieben wird der Fall einer 33-jährigen Patientin, die in der 39. Schwangerschaftswoche mit rezidivierender Epistaxis in unserer HNO-Ambulanz vorstellig wurde. Es zeigte sich endonasal links eine livide, die Nase subtotal verlegende Raumforderung, die zur Verformung der äußeren Nase führte. Eine externe Biopsie erbrachte keinen Hinweis auf Malignität. In der postpartal durchgeführten Computertomographie der Nasennebenhöhlen zeigte sich eine das knorpelige Nasenseptum destruierende Raumforderung. Es erfolgte eine endoskopische Resektion des Befundes unter Erhalt des klinisch nicht arrodierten Nasenseptumknorpels. Die histopathologische Aufarbeitung erbrachte den Nachweis eines kapillären Hämangioms, das angesichts des Auftretens in der Schwangerschaft als *Granuloma gravidarum* eingeordnet wurde.

## Abstract (en)

This article presents the case of a 33-year-old woman who consulted the authors’ ENT clinic in the 39th week of pregnancy with recurrent epistaxis. A livid endonasal mass was found on the left side, subtotally displacing the nose and leading to deformation of the external nose. External biopsy provided no indications of malignancy. Postpartum CT of the paranasal sinuses revealed a mass destroying the cartilaginous nasal septum. Endoscopic resection of the finding was performed with preservation of the clinically sound nasal septal cartilage. Histopathological examination revealed a capillary hemangioma, which was classified as granuloma gravidarum due to its occurrence during pregnancy.

## Falldarstellung

### Anamnese

Wir berichten über eine 33-jährige Patientin in der 39. Schwangerschaftswoche, die sich mit rezidivierender Epistaxis linksseitig in unserer HNO-Ambulanz vorstellte. Die Blutungsepisoden seien nach jeweils 30 min spontan sistiert.

### Klinischer Befund

In der HNO-ärztlichen Untersuchung zeigte sich eine die linke Nasenhaupthöhle subtotal verlegende, livide verfärbte Raumforderung. Aufgrund der Größe der Raumforderung kam es zu einer Deformierung des linksseitigen Nasenflügels. Die rechte Nasenhaupthöhle war nicht affektiert. Bedingt durch die Ausdehnung des Befundes und die Druckdolenz war die Identifikation des Ansatzes der Raumforderung nicht möglich. Der übrige HNO-ärztliche Befund zeigte keine Auffälligkeiten. In der sonographischen Untersuchung der Halsweichteile zeigten sich ebenfalls keine Auffälligkeiten, insbesondere keine pathologischen Lymphknoten.

### Therapie und Verlauf

Im Rahmen der initialen Vorstellung wurde bei unklarer, anamnestisch rasch progredienter Raumforderung der linken Nasenhaupthöhle nach konsiliarischer Rücksprache mit der Geburtshilfe eine Biopsie in Lokalanästhesie entnommen. Im Rahmen dessen kam es zu einer ausgeprägten Blutung aus der stark vaskularisierten Raumforderung, die erst nach Applikation lokaler Vasokonstringenzien, bipolarer Koagulation und Auflage eines lokalen Hämostyptikums zum Stillstand kam.

**Figure 1:**
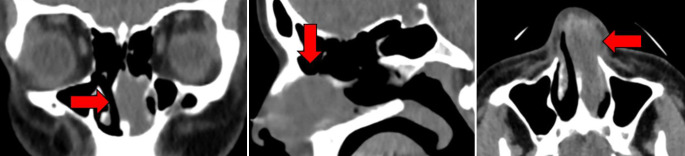

Nach komplikationsloser Entbindung fünf Tage nach Erstvorstellung in unserer Abteilung erfolgte zur weiterführenden Abklärung der Raumforderung die Durchführung einer Computertomographie der Nasennebenhöhlen. Hier zeigte sich eine weichteildichte 26 × 16 × 29 mm messende Raumforderung der linken Nasenhaupthöhle mit unscharfer Begrenzung zum Nasenflügel und Ballonierung des Nasenseptums zur Gegenseite (Abb. 1).

Bei der Kontrolle in unserer Ambulanz drei Tage postpartal zeigte sich ein deutlicher Progress der Raumforderung, die die äußere Nase zunehmend verformte und das Nasenseptum rechtskonvex deviierte. Die histopathologische Aufarbeitung der in lokaler Anästhesie entnommenen Biopsie ergab den Verdacht auf ein Angiofibrom, verblieb jedoch ohne Anhalt auf Malignität.

Aufgrund des atypischen histopathologischen Befundes für eine 33-jährige Patientin und der klinisch raschen Progredienz wurde die umgehende Indikation zur endoskopischen Exploration und Resektion der Raumforderung gestellt, die komplikationslos in Intubationsnarkose durchgeführt wurde.

Intraoperativ stellte sich eine nachhaltige Änderung des Lokalbefundes dar. Nach Absaugen von reichlich Koagel zeigte sich die ehemals prall-elastische, stark vaskularisierte Raumforderung nun stark atroph und nur wenig blutend. Es zeigte sich zudem, dass die Raumforderung an der septalen Nasenschleimhaut links gestielt war. Nach Umschneidung des Stiels und epiperichondrialer Dissektion zur Schonung des Nasenseptums konnte die Raumforderung in toto reseziert werden. Der Nasenseptumknorpel war darüber hinaus intakt. Es erfolgte eine Einsendung des Resektats zur Schnellschnittdiagnostik, aus der sich lediglich polypöses Gewebe ohne Anhalt für Malignität ergab. Aufgrund des histopathologischen Vorbefundes eines Angiofibroms erfolgte zudem eine Infundibulotomie mit Exploration des vorderen Ethmoids. Zum Schutz des freigelegten Nasenseptumperichondriums erfolgte die Einlage von Doyle-Splints. Aufgrund der ehemals ausgeprägten Blutungsneigung erfolgte zudem die Einlage einer einseitigen, fadenarmierten Nasentamponade.

**Figure 2:**
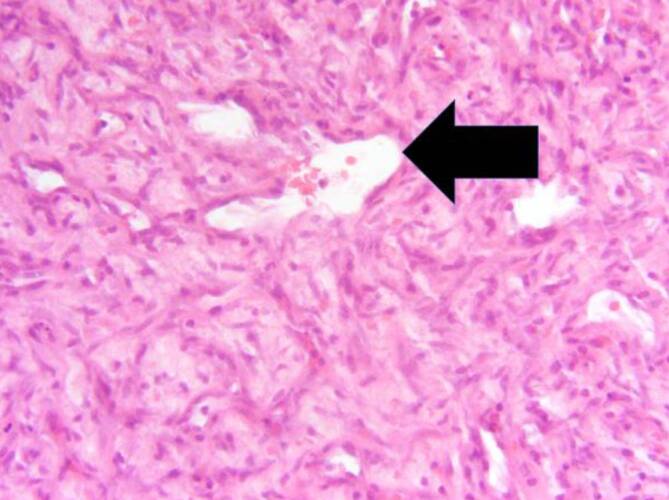

Im abschließenden histopathologischen Befund ergab sich die Diagnose eines lobulären kapillären Hämangioms mit überkleidendem Epithel mit Plattenepithelmetaplasie ohne Dysplasie. Dergestalt in der Schwangerschaft auftretende lobuläre kapilläre Hämangiome werden auch als *Granuloma gravidarum* bezeichnet (Abb. 2).

In den postoperativen Verlaufskontrollen zeigte sich eine stadiengerechte Abheilung des Schleimhautdefekts am Nasenseptum ohne Ausbildung einer Perforation sowie ein reizlos abgeheiltes Infundibulum ethmoidale.

## Diskussion

Unilaterale Raumforderungen der Nasenhaupt- und Nasennebenhöhlen sollten aufgrund der Möglichkeit einer malignen Genese grundsätzlich kurzfristig histopathologisch abgeklärt werden. Neben entzündlichen und malignen Prozessen stellen vaskuläre Raumforderungen, insbesondere bei männlichen jugendlichen Patienten, eine mögliche Differenzialdiagnose dar. Lobuläre kapilläre Hämangiome (LKH) sind benigne vaskuläre Tumoren, die klinisch initial als kleine rote Papeln erscheinen, exophytisch wachsen und eine Größe von bis zu mehreren Zentimetern erreichen können [4, 12]. Ein polypoid anmutendes oder auch ein gestieltes Erscheinungsbild werden als typisch angesehen, während breitbasige Formen selten sind [9]. Die alternativ verwendete Bezeichnung als *Granuloma pyogenicum* ist irreführend, da LKH nicht auf dem Boden einer Inflammation entstehen und es sich histopathologisch nicht um Granulome handelt [11]. Die Ätiologie der nasalen LKH ist nicht abschließend geklärt. Als assoziierte Risikofaktoren werden neben traumatischen Läsionen, angiogener hormoneller Stimulation auch Gravidität und die Einnahme oraler Kontrazeptiva angesehen [6].

LKH treten sowohl kutan als auch mukosal auf. Mukosale LKH betreffen vorwiegend Zunge und Gingiva, während die nasalen Schleimhäute, die Konjunktiven sowie Cervix und Vagina seltener betroffen sind [9]. Nasale LKH manifestieren sich vorwiegend am Nasenseptum, im Nasenvorhof oder an der unteren Nasenmuschel und werden durch unspezifische Symptome wie unilaterale Epistaxis oder Nasenatmungsbehinderung infolge der Okklusion der Nasenhaupthöhle klinisch apparent [1, 8, 10]. Inwieweit nasale LKH zur Rezidivbildung neigen, ist unklar. So berichten Puxeddu et al. in einer retrospektiven Analyse an einem Kollektiv von 40 Patient*innen nach endoskopischer Resektion von sinunasalen LKH und einem durchschnittlichen Follow-up von 53 Monaten, dass sich kein Rezidiv zeigte [8]. Konträr dazu berichten Smith et al. an einem Kollektiv von 34 Patient*innen von einer mit 42 % hohen Rezidivrate [10]. In dieser Studienpopulation trat in der Subgruppe der Schwangeren sogar in 3 von 5 Fällen ein Rezidiv auf. In der Schwangerschaft auftretende LKH werden als *Granuloma gravidarum* bezeichnet, sind histopathologisch nicht von anderen LKH zu unterscheiden [7] und treten oral und nasal in 2–5 % aller Schwangerschaften auf [3]. Eine histopathologische Abklärung des Befundes sollte stets zum Ausschluss eines Malignoms schon während der Schwangerschaft in Lokalanästhesie und in Absprache mit der betreuenden Geburtshilfe erfolgen. Bei Nachweis eines LKH respektive *Granuloma gravidarum* kann dann der Spontanverlauf während der Schwangerschaft abgewartet werden. Nach Ende der Schwangerschaft und des damit verbundenen Wegfalls der hormonellen Stimulation zeigen sich LHK in der Regel spontan regredient [5, 12]. Kommt es bei Schwangeren zu rezidivierender oder Hb-relevanter Epistaxis, sollte die Entfernung bereits in der Schwangerschaft erwogen werden. Bei ausbleibender Regredienz des Befundes zwei Monate post partum sollte eine Entfernung favorisiert werden [3]. Die operative Entfernung eines nasalen LKH sollte die Dissektion der Schleimhautbasis des Befundes beinhalten, und bei besonders ausgedehnten Befunden sollte die präoperative Embolisation erwogen werden [2, 3].

## Fazit für die Praxis

- Nasale lobuläre kapilläre Hämangiome treten vermehrt in der Schwangerschaft auf und neigen postpartal zur spontanen Regredienz.
- Bei ausgedehnten Befunden sollte dem Risiko des Auftretens von Hb-relevanter Epistaxis Rechnung getragen werden und die präpartale Exzision erwogen werden.
- Nachsorgeuntersuchungen erscheinen trotz bislang nicht beschriebener maligner Transformation aufgrund der unklaren Rezidivneigung sinnvoll.

## References

[1] Chi TH, Yuan CH, Chien ST (2014). Lobular capillary hemangioma of the nasal cavity: a retrospective study of 15 cases in taiwan. Balkan Med J.

[2] Choudhary S, Mackinnon CA, Morrissey GP (2005). A case of giant nasal pyogenic granuloma gravidarum. J Craniofac Surg.

[3] Delbrouck C, Chamiec M, Hassid S (2011). Lobular capillary haemangioma of the nasal cavity during pregnancy. J Laryngol Otol.

[4] Lee HM, Lee SH, Hwang SJ (2002). A giant pyogenic granuloma in the nasal cavity caused by nasal packing. Eur Arch Otorhinolaryngol.

[5] Loes S, Tornes K (2008). Misinterpretation of histopathological results as an important risk factor for unneeded surgery—case report of a “near miss” event in a pregnant woman. Patient Saf Surg.

[6] Lopez A, Tang S, Kacker A (2016). Demographics and etiologic factors of nasal pyogenic granuloma. Int Forum Allergy Rhinol.

[7] Mcshane DP, Walsh MA (1988). Nasal granuloma gravidarum. J Laryngol Otol.

[8] Puxeddu R, Berlucchi M, Ledda GP (2006). Lobular capillary hemangioma of the nasal cavity: a retrospective study on 40 patients. Am J Rhinol.

[9] Sarwal P, Lapumnuaypol K (2022). Pyogenic granuloma.

[10] Smith SC, Patel RM, Lucas DR (2013). Sinonasal lobular capillary hemangioma: a clinicopathologic study of 34 cases characterizing potential for local recurrence. Head Neck Pathol.

[11] Tamaki A, Babajanian E, D’anza B (2017). Lobular capillary hemangiomas: case report and review of literature of vascular lesions of the nasal cavity. Am J Otolaryngol.

[12] Zarrinneshan AA, Zapanta PE, Wall SJ (2007). Nasal pyogenic granuloma. Otolaryngol Head Neck Surg.

